# Treatment with the PI3K inhibitor buparlisib (NVP-BKM120) suppresses the growth of established patient-derived GBM xenografts and prolongs survival in nude rats

**DOI:** 10.1007/s11060-016-2158-1

**Published:** 2016-06-09

**Authors:** I. A. Netland, H. E. Førde, L. Sleire, L. Leiss, M. A. Rahman, B. S. Skeie, H. Miletic, P. Ø. Enger, D. Goplen

**Affiliations:** 1 Oncomatrix Research Lab, Department of Biomedicine, University of Bergen, Bergen, Norway; 2 Neuro Clinic, Haukeland University Hospital, Bergen, Norway; 3 Department of Clinical Medicine, K1, University of Bergen, Bergen, Norway; 4 Department of Biomedicine, Kristian Gerhard Jebsen Brain Tumour Research Center, University of Bergen, Bergen, Norway; 5 Department of Pathology, Haukeland University Hospital, Bergen, Norway; 6 Department of Neurosurgery, Haukeland University Hospital, Bergen, Norway; 7 Department of Oncology, Haukeland University Hospital, Bergen, Norway

**Keywords:** Glioblastoma, Brain tumours, PI3K, Proliferation, Targeted therapy, Patient-derived xenograft

## Abstract

Glioblastomas (GBMs) are aggressive brain tumours with a dismal prognosis, despite combined surgery, radio- and chemotherapy. Close to 90 % of all GBMs harbour a deregulated PI3K pathway, which is essential in regulating central cellular functions such as proliferation, cell growth, motility and survival. Thus, PI3K represents a potential target for molecular therapy in GBM. We investigated the anti-tumour efficacy of the PI3K inhibitor buparlisib (NVP-BKM120) in GBM cell lines in vitro and in vivo, when treatment was initiated after MRI-confirmed tumour engraftment. We found that buparlisib inhibited glioma cell proliferation in a dose dependent manner, demonstrated by MTS assay, manual cell count and BrdU incorporation. A dose dependent increase in apoptosis was observed through flow cytometric analysis. Furthermore, by immunocytochemistry and western blot, we found a dose dependent inhibition of Akt phosphorylation. Moreover, buparlisib prolonged survival of nude rats harboring human GBM xenografts in three independent studies and reduced the tumours’ volumetric increase, as determined by MRI. In addition, histological analyses of xenograft rat brains showed necrotic areas and change in tumour cell nuclei in buparlisib-treated animals. The rats receiving buparlisib maintained their weight, activity level and food- and water intake. In conclusion, buparlisib effectively inhibits glioma cell proliferation in vitro and growth of human GBM xenografts in nude rats. Moreover, the compound is well tolerated when administered at doses providing anti-tumour efficacy. Thus, buparlisib may have a future role in glioma therapy, and further studies are warranted to validate this compound for human use.

**Electronic supplementary material:**

The online version of this article (doi:10.1007/s11060-016-2158-1) contains supplementary material, which is available to authorized users.

## Introduction

Glioblastoma (GBM) is the most aggressive primary brain tumour [1] with a median survival of 14.6 months. Less than 10 % of patients survive 5 years after diagnosis [2]. The dismal prognosis for GBM patients, despite multimodal treatment calls for new therapeutic strategies.

Phosphatidylinositol 3-kinases (PI3Ks) are lipid kinases involved in cell proliferation, growth, apoptosis, DNA repair, angiogenesis, differentiation, motility and survival [3]. PI3Ks act as intermediate signaling molecules, involved in cell signaling pathways such as the PI3K/Akt/mTOR [4]. In normal cells, the PI3K activity is tightly regulated. Deregulation of the PI3K signaling pathway is common in cancer, including GBM [5]. Brennan and colleagues reported mutations in PI3K in 25.1 % of GBMs, whereas 89.6 % of GBMs had at least one alteration in the PI3K pathway including its other components, such as RTKs and PTEN [6]. Activation of this pathway is associated with increasing tumour grade and decreasing overall survival in gliomas [7]. The initial step in the PI3K pathway is PI3K-mediated phosphorylation of membrane bound phosphatidylinositols, generating second messengers (PIP3, PI 3,4-bisphosphate), which subsequently trigger a signaling cascade eventually activating multiple effector kinase pathways, such as the mTOR, ERK1/2, p38 MAPK, NF-kappa-B, and JNK/SAPK [4]. A key event in this cascade is binding of PIP3 to Akt, which is activated by phosphorylation at S473 and T308 [8]. The activation of Akt is assumed to be responsible for its growth-promoting and anti-apoptotic effects in tumour cells [4].

Given its role as a major regulator of multiple aspects of tumour cell behavior, PI3K has become a major target for drug design. Several small molecule PI3K inhibitors have been developed and are currently under pre-clinical and clinical assessment [4]. However, since the blood–brain-barrier (BBB) limits availability of drugs to the central nervous system (CNS), brain tumours are generally not considered attractive malignancies for initial drug screenings. For the same reason, few animal studies and hardly any patient trials have been published regarding the anti-tumour efficacy of PI3K inhibitors in gliomas.

Buparlisib (NVP-BKM120) is an orally bioavailable, small molecule compound with potent, pan-class I PI3K inhibitory capability against the p110-α, -β, -δ, and -γ catalytic subunit isoforms at IC50 doses in a micromolar range [9]. Since buparlisib penetrates the BBB [9], it represents an attractive candidate for targeted glioma therapy. Previously, Koul et al. reported inhibition of cell line derived brain tumour growth in SCID mice using buparlisib administered 4 days following implantation of the tumour cells. In the clinical setting, therapy is usually initiated after the tumour is confirmed by MRI and biopsy. Here we demonstrate efficacy of buparlisib in treatment of patient derived in vivo passaged GBM biopsy material in the experimental setting resembling the usual clinical situation with tumour confirmed by MRI. The in vivo passaged GBM biopsies develop tumours that histologically closely resemble the GBM in situ [10]. The aim of the present study was to evaluate the efficacy of buparlisib treatment, initiated after confirming tumour engraftment by MRI in a clinically relevant brain tumour animal model [10].

## Materials and methods

### Cell line and culturing

Cells were maintained in humid incubators at 37 °C and 5 % CO_2_. The U87 cell line was purchased from American Type Culture Collection (ATCC, Manassas, VA, USA) and cultured in DMEM (Sigma-Aldrich, St. Louis, MO, USA) supplemented with 10 % fetal bovine serum, 3.2 % non-essential amino acids, 100 units/mL Penicillin/Streptomycin, 400 mol/L l-glutamine (all Lonza, Cologne, Germany) and 0.005 mg/mL Plasmocin (InvivoGen, San Diego, CA, USA). Prior to animal implantation, spheroids of U87 cells were prepared; 1000 cells were centrifuged at 2250 rpm for 30 min in 96-well plates with conical bottom, containing 0.05 % methylcellulose. After 7 days incubation, each spheroid contained 17,000 cells. Three spheroids (51,000 cells) were implanted in each animal. For in vitro assessment of buparlisib efficacy, a 10 mM stock solution was prepared by dissolving buparlisib [kindly provided by Novartis (Basel, Switzerland)] in 100 % DMSO (Sigma-Aldrich).

### Patient tumour material and culturing

Tumour biopsy tissue was obtained from the operating theatre, Haukeland University Hospital, Bergen, after approval from the regional Ethical Board and consent from patients. Xenograft spheroids (P3) were prepared from serially passaged GBM biopsy tissue (as described by Wang [10]) and cultured as described by Bjerkvig [11]. Prior to animal implantation, the spheroid material was enzymatically dissociated at 37 °C by trypsin-EDTA and DNase (Roche, Basel, Switzerland) and resuspended in sterile PBS with 25 mM glucose (both Sigma-Aldrich). 100,000 cells were implanted in each animal. For in vitro experiments, the spheroid material was grown in monolayer and maintained in NB medium (Thermo Fisher Scientific Corporation, Carlsbad, CA, USA) with the addition of 32 IE/mL heparin, 20 ng/mL bFGF and 20 ng/mL EGF (Millipore Corporation, Billerica, MA, USA).

### Cell viability (MTS assay)

Cells were seeded in 96-well plates 24 h prior to treatment with buparlisib at concentrations ranging from 0 to 10 µM. After 72 h of exposure, the cells were subjected to MTS viability assay according to the manufacturer’s protocol (CellTiter 96® AQ_ueous_ One Solution Cell Proliferation Assay, Promega, Madison, WI, USA). Dose response curves for determination of IC50 values were generated in GraphPad Prism 6 (GraphPad Software Inc., La Jolla, CA, USA).

### Cell count

Cells were seeded 24 h prior to exposure of buparlisib for 72 h, before enzymatically detached by Trypsin–EDTA solution (Sigma-Aldrich), and manually counted in a Burker chamber haemocytometer.

### BrdU-pulsing

Cells exposed to buparlisib for 72 h were treated with 10 µM BrdU (Sigma-Aldrich) for 45 min at 37 °C. They were detached using a cell scraper, washed with 1xPBS and resuspended to 1 × 10^5^ cells/mL. 100 µL cell suspension was loaded into individual sample chambers and centrifuged in a Shandon CytoSpin centrifuge (Thermo Fisher Scientific, Wilmington, DE, US) at 800 rpm for 3 min. Immobilized cells were fixed (ICC-section) and subsequently subjected to immunocytochemistry, imaging and quantification. For each slide, three randomly picked areas (832 × 665.6 µM, 554 mM^2^) were selected for quantification. FITC stained cells and total number of cells was manually counted, and the proportion of FITC positive cells was calculated.

### Annexin V/propidium iodide (PI) apoptosis assay

Cells were stained with the Annexin V apoptosis assay according to the manufacturer’s protocol (Thermo Fisher Scientific). Samples were analysed on Accuri C6 (BD Biosciences) flow cytometer.

### Immunocytochemistry (ICC)

Cells were fixed in 4 % paraformaldehyde (Thermo Fisher Scientific Corporation) for 10 min, permeabilized by 0.5 % Triton X-100 (Sigma-Aldrich) in PBS for 4 min and incubated with blocking buffer [0.5 % BSA (Sigma-Aldrich) in PBS] for 15 min. Cells were incubated with primary antibodies O/N at 4 °C. The primary antibodies used were total Akt, pAkt S473, pAkt T308 (all Cell Signaling Technology, Danvers, MA, USA), and BrdU (Abcam, Cambridge, UK) together with DNase (Roche). Following incubation, cells were washed in PBS and incubated with secondary antibodies for 45 min at 37 °C. The secondary antibodies used were FITC-conjugated [S473; goat anti-rabbit (Southern Biotechnologies Associates Inc., Birmingham, AL, USA)] or AF555-conjuagted [Total Akt; goat anti-mouse (Thermo Fisher Scientific)]. Cells were mounted with Vectashield mounting medium with DAPI (Vector Laboratories, Burlingame, CA, USA). Fluorescent images were obtained with a Nikon TE2000-E microscope (Nikon Corporation, Tokyo, Japan).

### Immunoblotting (western blot)

Cells and tissue were harvested in kinexus buffer [20 mM MOPS, 5 mM EDTA, 2 mM EGTA, protease and phosphatase inhibitor tablets (Roche)], followed by sonication 3 × 5 s. Protein concentrations were determined by Pierce BCA Protein Assay Kit (Thermo Fisher Scientific Corporation). 20 µg sample was mixed with LDS sample loading buffer and sample reducing agent (both NuPAGE, Thermo Fisher Scientific Corporation) and incubated at 70 °C for 10 min. Samples were run on a pre-cast NuPAGE SDS-gel (Thermo Fisher Scientific Corporation) at 200 V for 60 min. Transfer to a nitrocellulose membrane was done at 30 V for 80 min. Following blocking in 5 % (w/w) Difco Skim milk powder (Becton, Dickinson and Company, Franklin Lakes, NJ, USA), in TBST for 1 h at RT, the membranes were incubated with primary antibody (total Akt, pAkt S473, pAkt T308) and β-actin (Santa Cruz Biotechnology Inc, Dallas, TX, USA) or GAPDH (Abcam) at 4 °C O/N. After washing, membranes were incubated with secondary antibodies [goat anti-mouse IgG-HRP (Santa Cruz Biotechnology Inc) and goat anti-Rabbit IgG (H+L) Cross Adsorbed Secondary Antibody, HRP conjugate (Thermo Fisher Scientific Corporation)] for 1.5 h at RT. For detection, Supersignal West Femto Maximum Sensitivity Substrate (Pierce Biotechnology, Rockford, IL, USA) was used, and chemiluminescent detection was obtained by a Fuji LAS 3000 Imager (Fuji Photo Film, Tokyo, Japan). Densitometric quantification was determined using ImageJ software (National Institutes of Health, Bethesda, MA, USA).

### Immunohistochemistry (IHC)

Paraffin-embedded sections from P3 xenograft rat brains were deparaffinised using xylene, 100 and 96 % ethanol, followed by antigen retrieval at 98 °C for 25 min in 10 mM citrate buffer, pH 6.0. Sections were treated with peroxidase and protein block (both Dako, Glostrup, Denmark) for 5 and 30 min, respectively. The primary antibody mouse anti-nestin (Millipore Corporation) was diluted to 1:1000 in Tris–BSA buffer, and added to the slides for 1 h at room temperature. Following washing with TBS-Tween, sections were incubated with anti-mouse secondary antibody (Dako) for 45 min at room temperature. Slides were developed with DAB chromogen (Dako) and counterstained with hematoxylin.

### Animals

Homozygous nude rats (rnu/rnu, Rowett) were used for the experiments. The animals were fed a standard pellet diet and provided water ad libitum, and kept in a pathogen free environment at a constant temperature and humidity with standard 12/12 h light and dark cycle. The experiment was approved by the Norwegian Animal Research Authority (Bergen, Norway). All animals were anaesthetized with 3 % isoflurane gas (Abbott Laboratories, Abbot Park, IL, USA) mixed with 50 % air and 50 % O_2_, and Marcaine (AstraZeneca, London, England) subcutaneously. Tumour implantation was performed as previously described [10]. Three animal studies were performed, and treatment started immediately after confirmed tumour take by MRI (U87 speroids; 10 days, P3 cell suspension; 21 days). Animals were randomly assigned to two different groups: (1) untreated controls and (2) 5 mg/kg buparlisib treatment (recommended by Novartis). Buparlisib suspension was prepared in 0.5 % methyl cellulose and 0.5 % Tween20 (both Sigma-Aldrich), while control group received vehicle only (0.5 % methyl cellulose and 0.5 % Tween20). Both groups were treated 5 days a week and received 10 mL/kg solution by oral gavage, using malleable oral dosing needles (Scanbur, Karlslunde, Denmark). Animals were weighed five times a week, inspected daily and euthanized by CO_2_ inhalation at the onset of symptoms.

### Magnetic resonance imaging (MRI)

MRI scans of all animal brains were obtained after tumour implantation to assess the tumour growth using a Bruker Pharmascan 7T small animal MRI (Bruker Biospin MRI GmnH, Ettingen, Germany). Axial T1- and T2-weighted images were obtained as previously described [10]. The tumour volumes at treatment and follow-up MRI were calculated in Gamma Plan (Elekta Instrument AB, Stockholm, Sweden).

### Statistical analysis

In vitro experiments were repeated three times and assessed by ANOVA with Tukey’s multiple comparions test, with a p value <0.05 considered significant. Kaplan–Meier survival curves were generated in GraphPad Prism 6 (GraphPad Software Inc.) for statistical analysis of the animal experiments. Median survival times for the treatment groups were compared using the log-rank test.

## Results

### Buparlisib inhibits cell growth and induces apoptosis of GBM cells

**Fig. 1:**
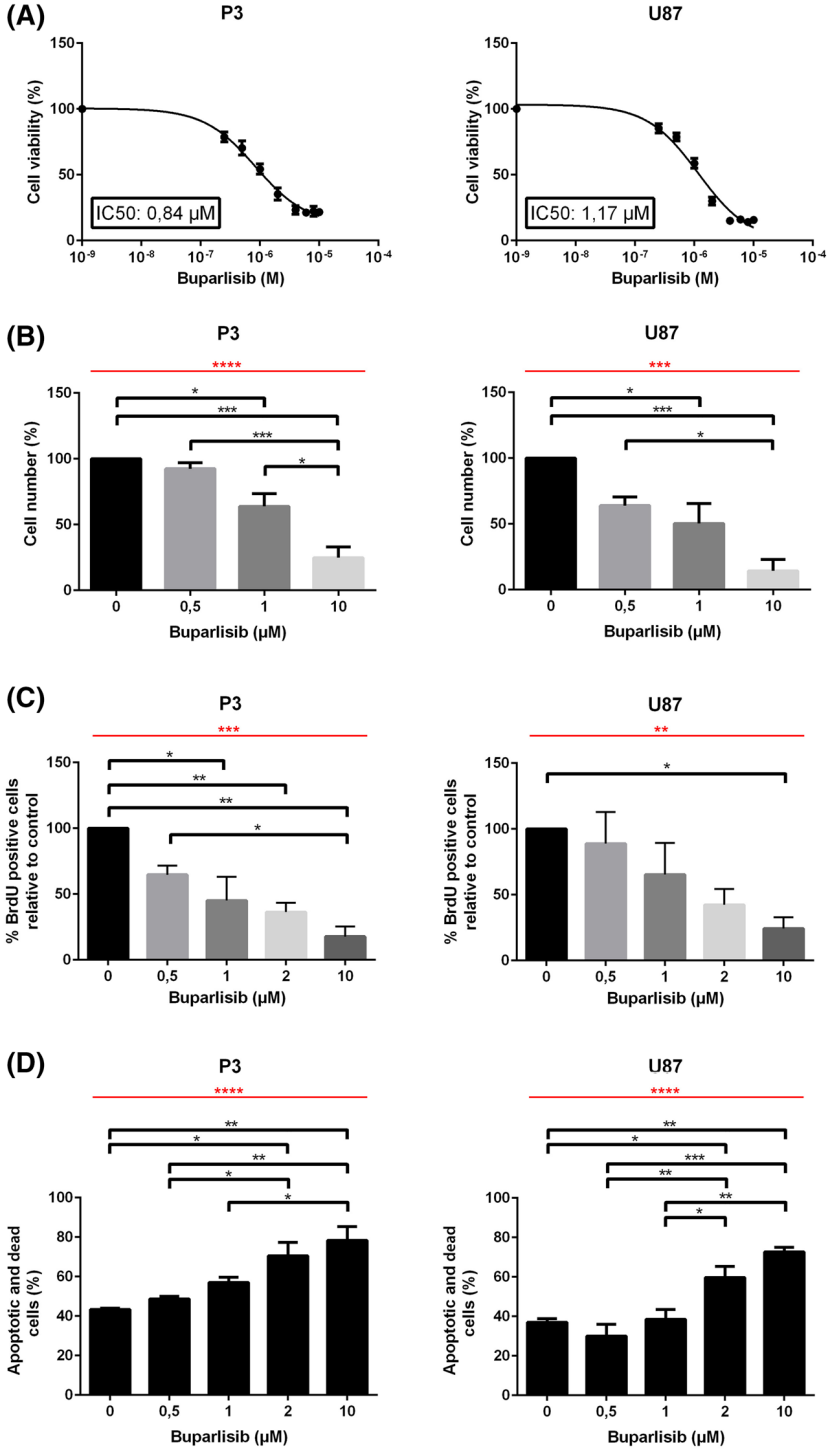
**a** IC50 doses of buparlisib for P3 (*left*) and U87 (*right*) glioma cells. **b** Relative cell number of P3 (*left*) and U87 (*right*) glioma cells exposed to buparlisib for 72 h. **c** Quantification of BrdU positive P3 (*left*) and U87 (*right*) glioma cells treated with buparlisib for 72 h with doses as indicated, and subsequently pulsed with BrdU. **d** Quantification of Annexin V- and PI-positive P3 (*left*) and U87 (*right*) glioma cells treated with buparlisib for 72 h with doses as indicated, and subsequently incubated with PI and Annexin V Alexa Fluor 488 conjugate. *Error bars* represent SEM. *Red bars* indicate p values for linear trends. All experiments were performed three times. *p < 0.05, **p < 0.01, ***p < 0.001

The cytotoxic potential of buparlisib on GBM cells was determined by exposing P3 GBM xenograft cells and U87 cells to buparlisib for 72 h, and assessing viability using the MTS assay. IC50 doses were found to be 1.17 and 0.84 µM for U87 and P3 cells, respectively (Fig. 1a). To test the side effects of the drug on non-tumor cells, glial cells from NOD/SCID mice brains were sorted and cultured in vitro, subsequently treated with 1 or 10 µM of buparlisib. After 72 h of exposure, treated cells and controls were analysed using the MTS assay. These data demonstrated that the metabolic activity of normal brain cells was significantly less affected than the P3 and U87 glioma cells (Supplementary Fig. 1). Furthermore, manual cell counting (Fig. 1b) showed that buparlisib reduced cell numbers in a dose dependent manner for both U87 and P3. In order to determine whether this reduction reflected increased cell death, decreased proliferation or both, untreated and buparlisib-treated cells were pulsed with the S-phase marker BrdU. Quantification of BrdU positive cells revealed a dose dependent decrease in cell proliferation (Fig. 1c), suggesting an anti-proliferate effect of buparlisib. The solvent (DMSO) did not show anti-proliferative effect (data not shown). Interestingly, flow cytometry of PI/Annexin V-stained cells demonstrated a dose-dependent increasing apoptosis upon buparlisib-treatment (Fig. 1d).

### Buparlisib inhibits phosphorylation of Akt in vitro

**Fig. 2:**
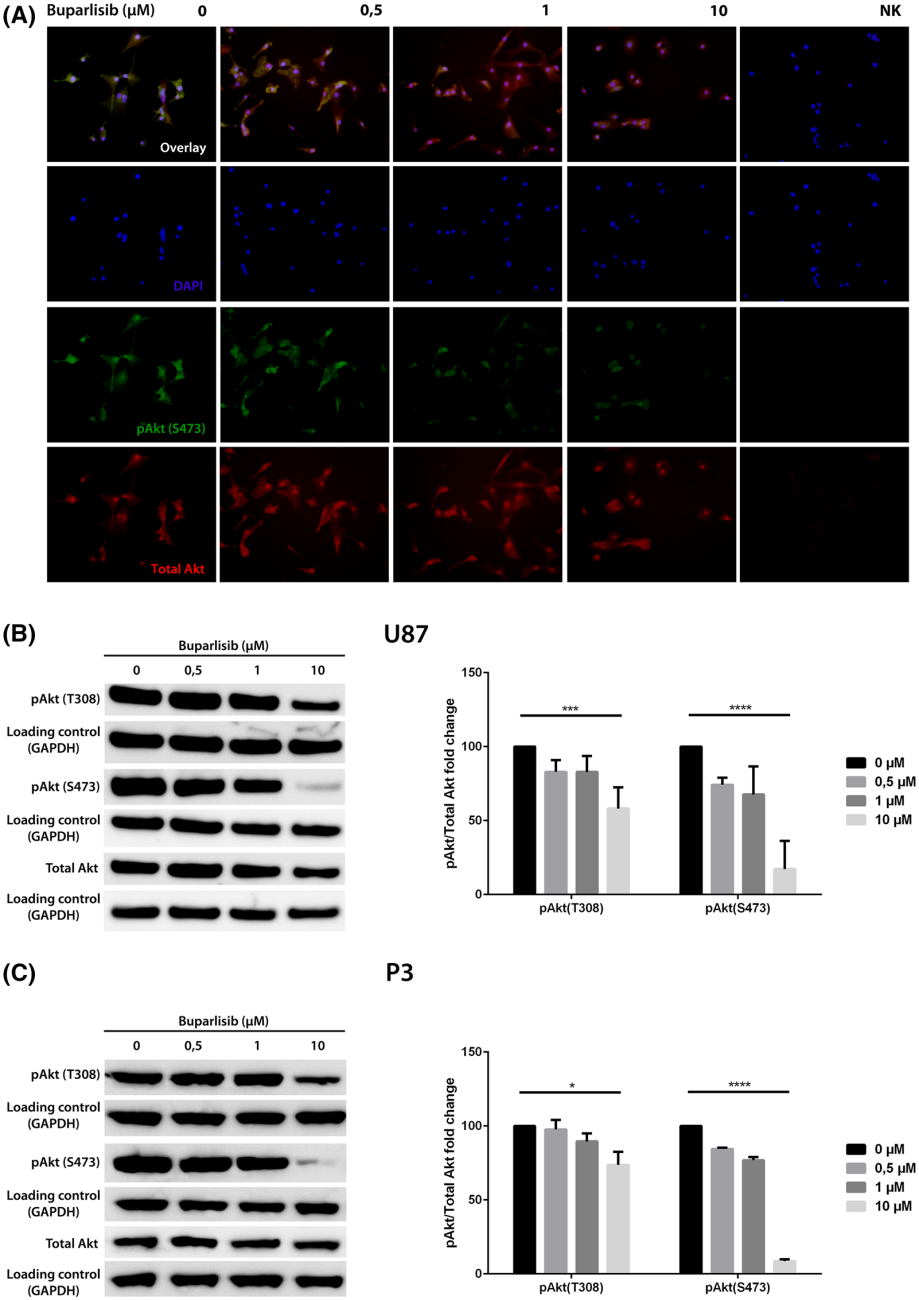
**a** Immunocytochemistry showing Akt phosphorylation in U87-cells at S473 after exposure to different concentrations of buparlisib for 72 h. *Upper panel* overlay image of Akt phosphorylated at site S473 (FITC, *green*) and total Akt (AP555, *red*) with DAPI nuclear counterstaining (*blue*). *Middle panel 1* DAPI nuclear staining (*blue*). *Middle panel 2* Akt phosphorylated at site S473 (FITC, *green*). *Lower panel* total Akt-levels (AP555, *red)*. **b** *Left* western blots showing levels of pAkt (T308), pAkt (S473) and total Akt in U87 cells exposed to buparlisib for 72 h. *Right* densitometric assessment of western blots, showing relative changes in phosphorylation. **c** *Left* western blots showing levels of pAkt (T308), pAkt (S473) and total Akt in P3 cells exposed to buparlisib for 72 h. *Right* densitometric assessment of western blots, showing relative changes in phosphorylation. Error bars represent SEM of three independent experiments. p values estimated represent linear trends. *p < 0.05, **p < 0.01, ***p < 0.001, ****p < 0.0001

The inhibitory effect of buparlisib on PI3K was shown by decreased activating phosphorylation of the PI3K downstream effector Akt. Akt is activated by phosphorylation of the amino acid residues threonine 308 (T308) and serine 473 (S473). ICC of U87 cells on coverslips, fixated after exposure of buparlisib for 72 h demonstrated a dose dependent reduction of Akt phosphorylation (Fig. 2a). Quantitative analysis was performed by western blot of lysates from U87 (Fig. 2b) and P3 (Fig. 2c) cells exposed to buparlisib in various concentrations and band intensities were assessed by densitometry. A dose dependent reduction of Akt phosphorylation at both sites (S473 and T308) was observed. Treatment with buparlisib did not alter the total level of Akt protein, indicating that the reduced level of phosphorylated Akt was caused by an inhibition of its phosphorylation and not by decrease of the Akt protein level.

### Buparlisib reduces tumour growth and prolongs survival in nude rats harbouring GBM xenografts

The anti-tumour efficacy of buparlisib in vivo was evaluated in a clinically relevant GBM animal model that uses intracerebrally engrafted in vivo passaged GBM xenografts derived from human biopsy material. This model reflects the growth pattern of human tumours in situ, including infiltration into the brain parenchyma and angiogenesis [10]. Three weeks after implantation of the tumour, MRI confirmed tumour take, and the animals were randomly assigned to two treatment groups: one receiving 5 mg/kg buparlisib for 5 consecutive days and 2 days rest, and one group receiving vehicle only (control).

**Fig. 3:**
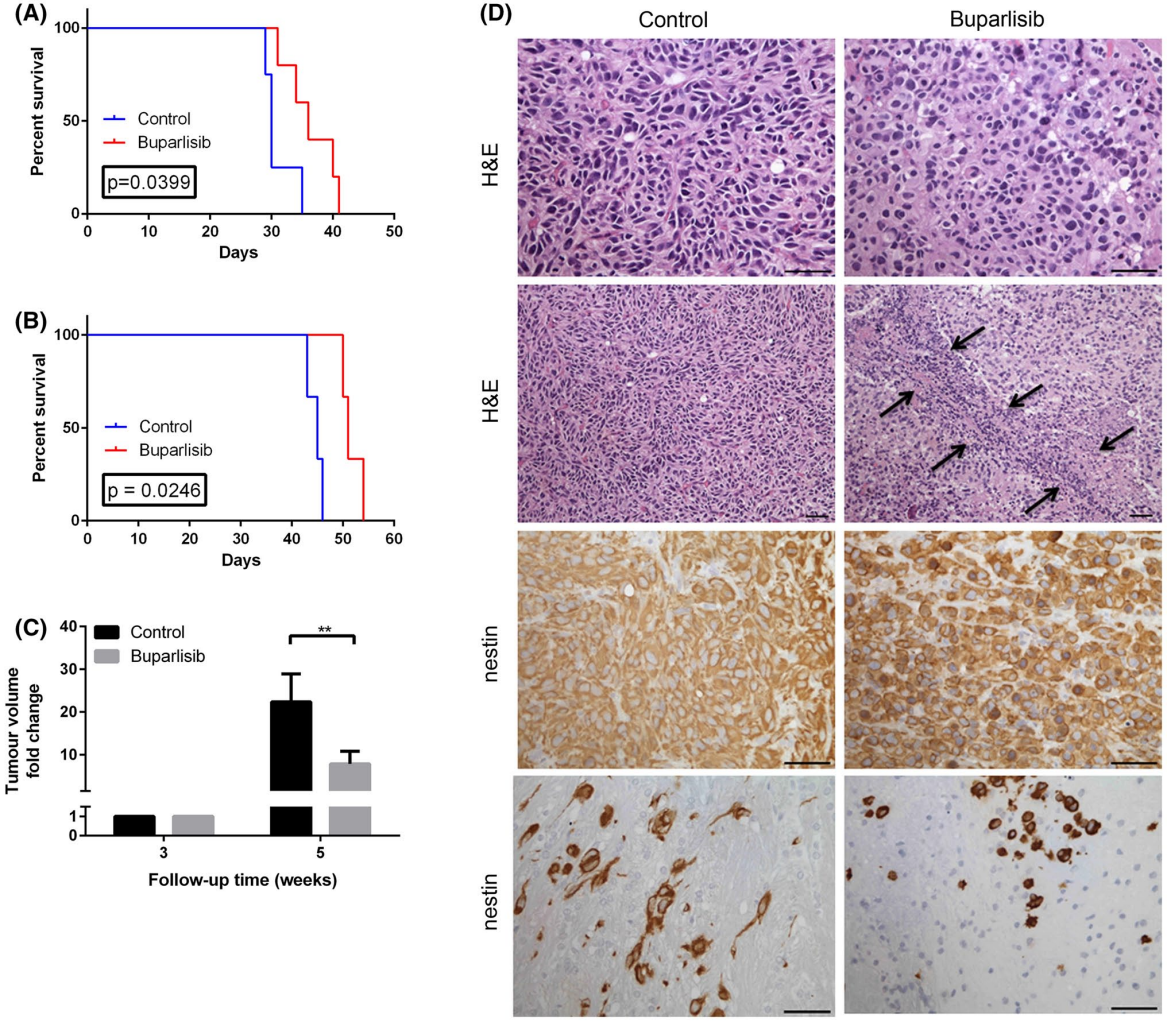
**a** Kaplan–Meyer survival curve for first study with nude rats carrying P3 xenografts (n = 9). **b** Kaplan–Meyer survival curve for the second study, with nude rats carrying P3 xenografts (n = 6). **c** MRI-based assessments of tumour volumes 3 weeks post implantation (treatment start) and 5 weeks post implantation. *p < 0.05. **d** H&E and nestin immunostained sections of treated and control tumors. *Arrows* indicate a necrotic tumour area. *Scale bars* 50 μm

In the first study, the median survival from implantation was 36 days (range 31–40 days) for the buparlisib-treated rats (n = 5), and 30 days (range 29–35 days) for the control rats (n = 4) (p = 0.039) (Fig. 3a). The survival benefit was confirmed in a second study with a median survival from tumour implantation of 51 days (range 50–54 days, n = 3) and 45 days (range 43–46 days, n = 3) in the treatment and control group respectively (p = 0.0246, Fig. 3b). MRI 2 weeks after treatment initiation revealed significantly smaller tumour volumes in the treatment group [20.2 mM^2^ versus 103.4 mM^2^ for the control group (Fig. 3c)]. Histology showed necrotic tumour areas in all four animals analysed from the treated group, while only one out of four animals from the control group showed a small necrotic tumour area (Fig. 3d). Furthermore, the density of tumour cell nuclei seemed to be reduced in the treated compared to control tumours. As the intermediate filament protein nestin is expressed in a very high fraction of tumour cells, we used it as a tumour marker. Immunostaining for nestin revealed that the morphology of tumour cells changed in the treated animals compared to controls. Tumour cells from the treated group showed a more epithelial-like phenotype while the tumour cells of the control tumours had a mesenchymal shape which was best visible in infiltrative tumour areas (Fig. 3d).

**Fig. 4:**
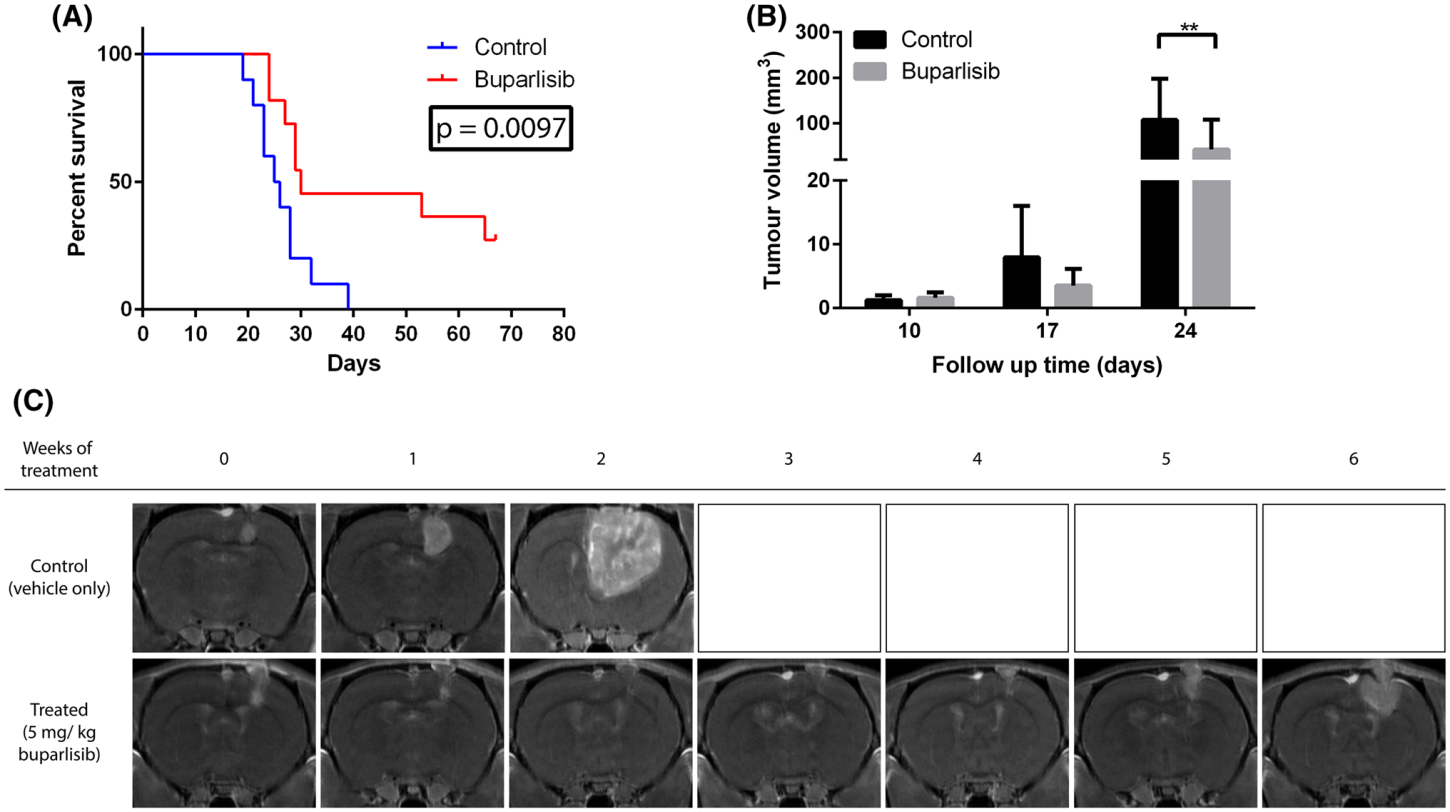
**a** Kaplan–Meyer survival curve for nude rats carrying orthotopic GBM cell line (U87) xenografts (n = 18). **b** MRI-based assessments of tumour volumes 10 days post implantation (treatment start) and 24 days post implantation. *p < 0.05 **c** T1-weighted magnetic resonance images (MRI) with contrast, from two representative rats; one from each group

To confirm previous findings in a larger study we stereotactically implanted U87 glioma spheroids and initiated buparlisib treatment after tumour engraftment was verified by MRI (10 days post implantation). In this study, the median survival in the control group (n = 9) was 25.5 days (range 19–39 days) from implantation versus 30 days (range 24–67 days) in buparlisib-treated rats (n = 9) (p = 0.0097, Fig. 4a). Notably, in the treatment group, some animals lived several weeks longer than untreated animals. MRI scans performed weekly revealed stable tumour volume, indicating prolonged progression free survival in the animals responding to the therapy (Fig. 4c). However, after the initial response, the tumours resumed growth and the animals developed symptoms.

### Buparlisib inhibits phosphorylation of Akt in vivo

**Fig. 5:**
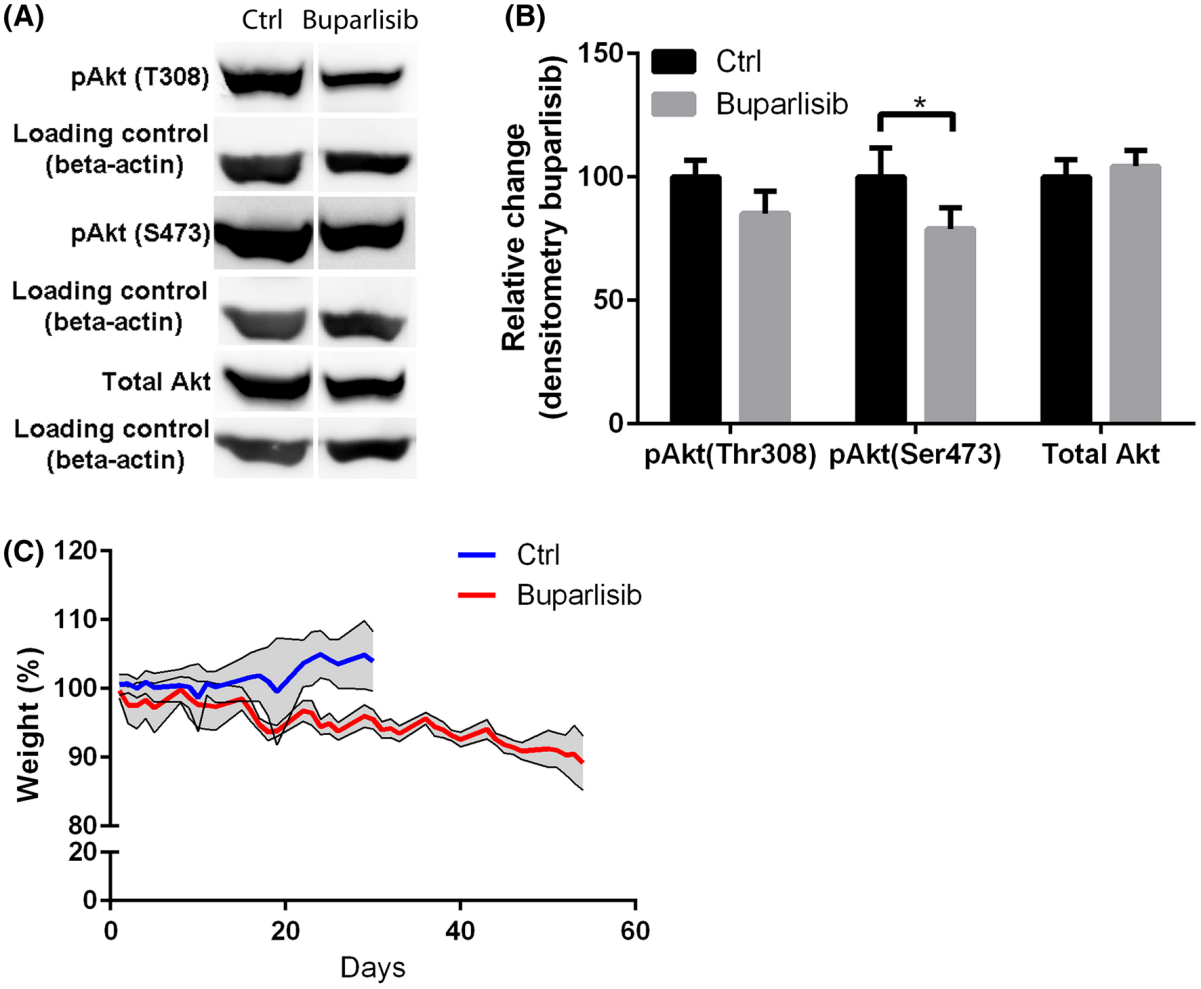
**a** Western blots showing levels of pAkt (T308), pAkt (S473) and total Akt in the tumours of one representative U87-xenografted rats from each group. Tumor material was collected 2–4 h post treatment when rats had reached humane endpoints. **b** Densitometric assessment of the western blot in (**a**), showing relative change in phosphorylation. *p < 0.05. **c** Weight measurements for the two groups

*Ex vivo* western blot analysis of tumour tissues from euthanized rats showed significant inhibition of Akt phosphorylation at S473. Phosphorylation at T308 was also reduced following treatment, although this difference was not significant. The total level of Akt protein was unchanged (Fig. 5a, b).

### Daily treatment with buparlisib in nude rats harbouring GBM xenografts is well tolerated

Daily inspection of the rats showed no change of activity or food and water intake. Throughout the experiment, both the treated and control animals showed stable body weight, although the treatment group displayed a slight weight reduction (Fig. 5c). The rats in the treatment group had temporary hair loss after 3 weeks of treatment. However, the animals exhibited hair re-growth while they were still on treatment. No severe side effects were observed.

## Discussion

We evaluated the anti-tumour efficacy of pan-PI3K inhibitor buparlisib on glioma. A dose dependent anti-proliferative effect of buparlisib in vitro, accompanied by inhibition of Akt phosphorylation at both serine 473 (S473) and threonine 308 (T308) was demonstrated. In vivo, buparlisib treatment led to significantly improved survival and reduced tumour volume. The compound seemed to be well tolerated by the animals, also during prolonged treatment over several weeks.

The observed in vitro anti-proliferative effect of buparlisib confirms previous findings, which include cell lines of glioma origin [9, 12], as well as other cell lines [9]. The ability of buparlisib to induce apoptosis as well as dose dependent reduction of Akt phosphorylation in vitro both at S473 and T308 is in line with previous reports [9, 13].

In our study, buparlisib demonstrated anti-tumour efficacy in an animal model employing patient-derived tumour material that was previously shown to mimic the growth of human gliomas in situ [14]. The three independent animal experiments confirmed previous reports of prolonged survival of animals with intracranial GBM xenografts [9]. However the efficacy of buparlisib in GBM therapy has not previously been studied using in vivo propagated patient-derived tumour material. Our results were further validated with a commonly used glioma cell line U87 [15]. However, we initiated buparlisib treatment up to 3 weeks following tumour implantation, after tumour engraftment was confirmed by MRI. Although, Koul et al. reported growth inhibition in an in vivo GBM model using buparlisib [9], they initiated treatment shortly after tumour implantation without prior confirmation of tumour engraftment. We believe our present data obtained in a model closely resembling the clinical setting where the relapsed tumour is detected by MRI, provide additional support for clinical validation of buparlisib for human GBMs. Interestingly, the observed anti-tumour efficacy of buparlisib extends beyond previous results, as one third of the animals experienced prolonged progression free survival and even slight reduction in tumour size for several weeks. However, the effect was temporary as the tumours eventually resumed growth. This reflects the palliative therapy of solid tumours, when tumour progression occurs after initial volume response and/or disease stabilization. Bradford et al. have also reported development of secondary resistance to buparlisib therapy. In endometrial cancer the resistance was mitigated by conventional chemotherapy [16].

*Ex vivo* analysis of the tumour samples obtained post mortem from treated animals demonstrated decreased phosphorylation of Akt, confirming that buparlisib does reach its intracranial target. This is in line with the published study of Koul and colleagues [9] and the previously reported BBB penetrance [9]. The histopathology of the tumours was altered in the treated animals indicating the therapeutic effect of the compound. Moreover, buparlisib seemed to be well tolerated by the animals, also over a prolonged exposure time. Similar observations have been made in phase I/II clinical studies of buparlisib [17–19].

Treatment resistance in cancer is commonly mediated through activation of the PI3K pathway [20]. Targeting PI3K may thus be a plausible strategy for treatment of GBM. Previous studies demonstrated that inhibition of PI3K increases the sensibility of GBM cells to doxorubicin treatment in vitro [21]. Buparlisib has demonstrated an additive effect when combined with temozolomide in glioma cells, and a synergistic effect when combined with MEK and HER2 inhibitors [9]. Here we show anti-tumour efficacy of a PI3K inhibitor alone. The secondary resistance to PI3K inhibitor might be attenuated by combining buparlisib with conventional chemotherapy. Currently, there are ongoing clinical trials assessing buparlisib monotherapy in recurrent GBM as well as of combination of buparlisib with other agents in newly diagnosed and recurrent malignant glioma.

In conclusion, we have shown that buparlisib has anti-tumour efficacy as a monotherapy in preclinical studies. Thus, we believe our data provide a rationale for clinical trials validating buparlisib as a single therapy in GBM patients.

## Electronic supplementary material

Below is the link to the electronic supplementary material.

Supplementary material 1 (DOC 146 KB)
