# Supplementary material for: Treatment with the PI3K inhibitor buparlisib (NVP-BKM120) suppresses the growth of established patient-derived GBM xenografts and prolongs survival in nude rats
Source: J Neurooncol. 2016 Jun 9;129:57–66. doi: 10.1007/s11060-016-2158-1 (PMC4972854; doi:10.1007/s11060-016-2158-1)
Supplement: Supplementary file 1 — Supplementary material 1 (DOC 146 KB) [file 11060_2016_2158_MOESM1_ESM.doc]

**Treatment with the PI3K inhibitor buparlisib (NVP-BKM120) suppresses the growth of established patient-derived GBM xenografts and prolongs survival in nude rats**

Netland IA^1*^, Førde HE^1*^, Sleire L^1^, Leiss L^1,2^, Rahman MA^1^, Skeie BS^3^, Miletic H^4,5^, Enger PØ^1,4,6^ and Goplen D^4,7^

^1^Oncomatrix Research Lab, Department of Biomedicine, University of Bergen, Bergen, Norway

^2^Neuro Clinic, Haukeland University Hospital. Bergen, Norway

^3^Department of Clinical Medicine, K1, University of Bergen, Bergen, Norway

^4^Kristian Gerhard Jebsen Brain Tumour Research Center, Department of Biomedicine, University of Bergen, Bergen, Norway

^5^Department of Pathology, Haukeland University Hospital, Bergen, Norway

^6^Department of Neurosurgery, Haukeland University Hospital, Bergen, Norway

^7^Department of Oncology, Haukeland University Hospital, Bergen, Norway

*These authors contributed equally

**Supplementary figures**

**
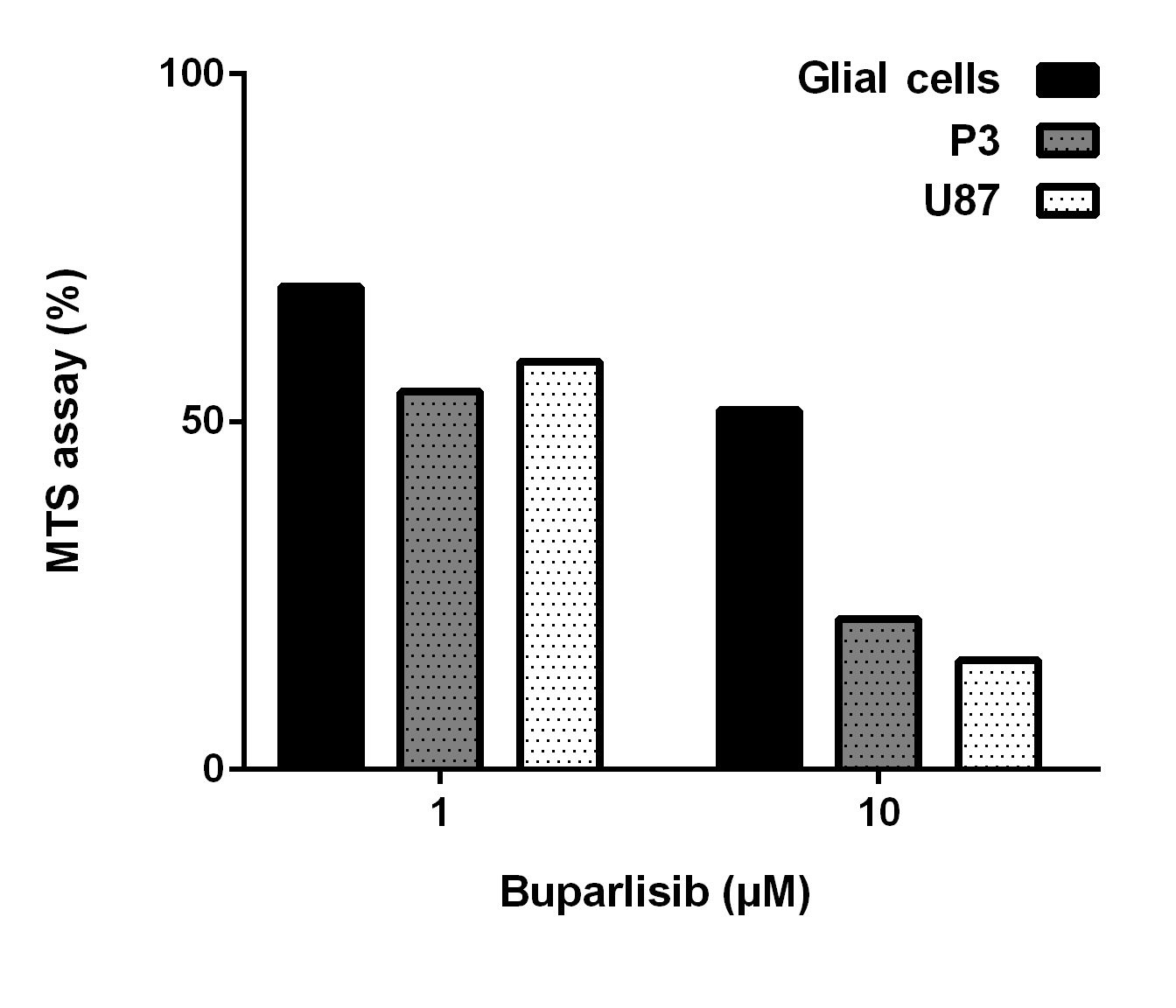
**

**Supplementary figure 1:** Toxicity testing of buparlisib on healthy brain cells compared to GBM cell lines U87 and P3. Healthy glial were sorted from a DsRed NOD/Scid mouse and treated with 0, 1 and 10 µM buparlisib for 72 h. Compared to U87 and P3 GBM cell lines, which were also treated with 0, 1 and 10 µM buparlisib for 72 h, the healthy brain cells are not as effected upon treatment as the highly proliferative tumor cell lines. ­

**MATERIAL AND METHODS**

**Toxicity testing on normal cells**

One DsRed NOD/Scid mouse was euthanized by CO_2_ and dislocation of the cervical vertebra. The brain was dissected out and dissociated and sorted to obtain healthy brain cells. The tissue was handled on ice, while cut into 1x1x1 mm pieces, washed once in HBSS (Gibco, Thermo Fisher Scientific, Waltham, Massachusetts, USA) and resuspended in 5 ml dissociation buffer (HBSS, 0,025mg/ml Liberase DH (Roche, Basel, Switzerland) and 160 KU/ml DNase (Worthington, Lakewood, New Jersey, USA)). Tissue was incubated in a shaking water bath at 37^o^C for a total of 90 minutes. Every 30 minutes, single cells were removed from the suspension and transferred to complete DMEM (see “Cell line and culturing”) for enzyme inactivation, after which fresh dissociation buffer­ was added for continued dissociation of the remaining tissue. Single cells were sequentially filtered through 100, 70 and 2x40µm cell strainers (BD, Erembodegem, Belgium) before sample was washed in 1xPBS. Single cells were separated from dead cells and debris by FACS Aria II (BD) based on SSC-A/FSC-A, FSC-w/FSC-A and DsRed+-A/FSC-A. 1x10^5^ acutely isolated cells were seeded/well in a 96 well plate and incubated over night. The following day, 0, 1 and 10µM BKM120 was added and cells incubated for another 72 hours before MTS assay was performed (see “Cell viability”).
